# Caring for affective subjects produced in intimate healthcare examinations

**DOI:** 10.1177/13634593211020072

**Published:** 2021-05-27

**Authors:** Jenny Gleisner, Ericka Johnson

**Affiliations:** Linköping University, Sweden

**Keywords:** affect, body, care, education, materiality

## Abstract

This article is about the feelings – affect – induced by the digital rectal exam of the prostate and the gynaecological bimanual pelvic exam, and the care doctors are or are not instructed to give. The exams are both invasive, intimate exams located at a part of the body often charged with norms and emotions related to gender and sexuality. By using the concept *affective subject*, we analyse how these examinations are taught to medical students, bringing attention to how bodies and affect are cared for as patients are observed and touched. Our findings show both the role care practices play in generating and handling affect in the students’ learning and the importance of the affect that the exam is (or is not) imagined to produce in the patient. Ours is a material-discursive analysis that includes the material affordances of the patient and doctor bodies in the affective work spaces observed.

## Introduction

The older man is sitting on the stretcher, his legs dangling over its edge incongruously. He is in his eighties, and at the urology clinic because he must urinate often and urgently, even though he claims he hardly drinks any fluid. The urologist, Hanna, comments to him that their patients often know where to find public toilets. This triggers a smile of mutual understanding in them both. The patient explains that he has an enlarged prostate and that he thinks it may cause these problems. Hanna states that his PSA^1^ is normal but that she will examine the prostate. She asks him to pull down his trousers and place himself on his left side on the stretcher with his back towards her. Hanna turns away to put on a plastic apron and gloves while the patient undresses, but the patient keeps on talking to her, which makes her turn towards him from time to time, even though she is trying to give him privacy as he undresses. At the start of the examination, they realize that the patient’s previous haemorrhoid surgery is making the digital rectal exam very painful, and Hanna applies generous amounts of topical anaesthesia crème. ‘This is completely normal,’ she says to the patient, intentionally reassuring him. But it is clear that he is uncomfortable.

The above fieldwork note succinctly articulates aspects of care that we are going to discuss – care which is much more complex than the mere practice of providing an exam in the name of health care. This article is about the feelings – affect – induced by the digital rectal exam of the prostate and the gynaecological bimanual pelvic exam, and the care doctors are or are not instructed to give during courses that teach the exams to medical students in Sweden. Focusing on care allows us to ask questions about subject positions, assumptions about the patient experience and the teaching of medical professionalism that is influenced by norms and values often associated with intersectional aspects of identity like sexgender, sexuality, age, class, race and health. In our analysis we will be focusing on sexgender and sexuality.^2^

Social aspects of care appear to differing degrees around intimate exams. They, as analytically generative spaces, in turn, allow for us to think about the structures and norms that shape medical health care provision and produce sexgendered doctors and patients.

Care, care work and emotional labour have been analysed in many different domains, especially as an essential element of certain professions (Hochschild, 1979; James, 1992), in particular health care (Allen, 2013). Much of this work has pointed to the tangled association of care with undercurrents of emotional attachment or domestic or familiar responsibilities (England and Dyck, 2011) and with its relationship to the body of the care giver and receiver (Twigg et al., 2011), particularly in the realm of nursing. Care is also discursively employed in the description of tasks often awarded low prestige and low pay and actioned as a motivation in providing these tasks in the face of low pay and low status (Stacey, 2005). The gendered aspect of care work is widely recognized, as is its relationship to class and race (Duffy, 2011; Meyer, 2000). Here one finds analysis of the frequent assignment of caring tasks to marginalized workers as well as analysis of how these tasks appear to be the target of labour process (re)organization (Cohen, 2011). This connection to labour is often seen in rhetoric around the promise of technological developments for care provision, such as telemedicine (Mort et al., 2006) and robotics (Sparrow, 2016; Wright, 2019), technology which would claim to complement or potentially replace care workers. Posthuman analysis of this care work (DeFalco, 2020) articulates how care is assigned qualities such as ‘good’ and ‘human’ but then questions what these assignments do.

These are all inspirational aspects of care that we take with us into our analysis. Yet, in this article, much of our analytical understanding of care is inspired by Science and Technology Studies (STS) and work on care practices that are situated and contextual (Mol, 2008; Singleton, 2012) and which can harm or reproduce vulnerabilities even as one attempts to care (Singleton and Mee, 2017). The work we have observed involves being responsible for the provider’s and the patient’s affective response to the vulnerabilities produced by particular examinations and the situated materialities of where and how that care is provided. The materiality of the intimate exams (and discourse, always, forever, being entangled with it) also speaks to concern for how care is received and provided. The material aspects of the exams are part of our analysis (the table, the lighting, the speculum or ultrasound), but so are elements that structure the discursive practices of medicine – the binary understanding of biological sex that has historically produced gynaecology and urology as separate fields with different norms and values – as well as structuring discursive concepts of femininity, masculinity and sexuality. These human and non-human actors, cultural figurations, discursive and physical elements are all entangled into the knot that we, through this analysis, hope to loosen and discuss. Therefore, we attend to care as a medical practice with affective discursive and material (social and embodied) aspects, which is why we attend to affective subjectivities, see below.

We will, at times, be using the term body to indicate the anatomical, biological, physical referent often assumed to be the object of medical knowledge construction. We see the term body as a categorizing tool, like sexgender, and in line with discussions that problematize the distinction between a physical body and a social subject (Johnson, 2017; Martin, 1991; Mol, 2002; Oudshoorn, 1994; Roberts, 2015; Wailoo, 1997; Zeiler, 2010). We employ body to reference an entangled understanding of patient subjectivities and medical anatomies that we are observing and note that it is often mediated by the material technologies of medical practice and their constellations, including the teaching methods and tools used to examine and represent it (Johnson, 2004, 2008; Dieckmann et al., 2019).

## Affective subjects and medicine

‘Affective subjectivity’ as a concept has found resonance in discussions about the relationship between psychiatrists and their patients, often in a therapeutic framework employing counter-transference and empathy. The term is used to connote, ‘the awareness of and reflection on our emotional responses and their influence on our work, and the development of a capacity for self-reflection and emotional attunement with our patients’ (Yakeley et al., 2014: 97; see also Sargeant, 2014). In these discussions, the affective subject is the provider of a health service, not the patient. The term recognizes the importance of a psychiatrist’s (or medical doctor’s) emotional response to their patients for the quality and type of care that the carer is able to provide. This is a conscious response and the term is used to draw attention to it.

But the concept of the affective subject can be equally useful in analysing how medical care practices create specific patient subjects, or at least collective imaginings of them. Used in this sense, it draws on Judith Butler’s discussion of performativity, which emphasizes the interplay between repetitive acts of being (or being done to/on, as in our case) and the performance of acceptable subjects. She writes,

We think of subjects as the kind of beings who ask for recognition in the law or in political life; but perhaps the more important issue is how the terms of recognition [. . .] condition in advance who will count as a subject and who will not (Butler, 2009: iv).

Using the concept *affective subject* brings attention to how patient bodies come into being through norms in medical practice, how bodies are encountered, observed and touched, through the material positionings of the patient and doctor bodies, as well as the feelings these examinations provoke. The concept also brings attention to how various pedagogical approaches consciously address how to handle one’s emotions in training situations, how to recognize and care for the feelings that particular patient encounters produce in a care provider’s body – or what they are expected to produce – and also how to display or hide them. This involves both acknowledging that one has been affected (in the way discussed by Sargeant, 2014; Yakeley et al., 2014) but also acknowledging the professional requirements of emotional control and learning the ‘proper’ feeling for the job (Gleisner, 2013; Gleisner and Siwe, 2020; Fineman, 2005; Hochschild, 1979) and learning how to be a ‘doctor’ (Becker et al., 2004 [1961]; Lindberg, 2009). Thus, we see affect and care as ‘tightly bound’ (Giraud and Hollin, 2016: 30) and intertwined with how patient subjectivities are produced in medical care practices.

Our use of the concept of affective subjects can also be traced to Haraway’s (1991, 1997) work on bodies and the way her ideas are engaged in Robert’s (2015) analysis of puberty. Hence, the concept affective subject shows how feelings are entangled with, and provoked by, bodies and how they come into being. Roberts uses the concept of an affective subject to explore how feelings (negative and positive) are often unreflectively entangled with a phenomenon. In her research, these are the practices and discourses of sexual development that produce affective subjects. In ours, they are the practices and discourses of intimate, invasive exams. Through its attention to affect and the subject, Roberts’ work, drawing on Haraway, inspires us to analytically explore those entanglements by trying to ‘loosen the knots’ of affect, care, materiality and medical practice (Haraway, 1997: 129). In Robert’s work, these knots joined early onset puberty to female sexuality, youth and anxiety, exploring ‘how these three intertwined elements – findings, feelings and figurations – articulate sexually developing bodies as bio-psycho-social’ (Roberts, 2015: 31). Roberts asserts that early onset puberty becomes a ‘gendered and gendering problem’, because of the attention given to female bodies and female sexuality (as problematic) in medicine, psychology and in public debates and compares this with the lack of research about male puberty. Reading along with her, we also loosen the knots that entangle the material-discursive practices in affective moments of care to articulate sexgender, sexuality and subject positions. Of note, however, is that most of these moments of affect are consciously reflected upon in our material; teaching the students the ‘proper way’ to deal with their emotions that are triggered by an exam, for example, but also teaching them to pay attention to the emotions that may be triggered in the patient. The affective subject of the care provider and the care receiver is conscientiously considered, but more so in one exam than in the other.

## Context, materials and methods

This article is based on a research project about how intimate, internal examinations are taught and practiced by medical students, and how the body is produced in the doctor-patient relationship.

We^3^ are comparing two exams where there are resemblances found in how they are performed and experienced: the digital rectal exam of the prostate and the bimanual pelvic exam, hereafter called the prostate exam and the pelvic exam. They are both invasive, intimate exams located at a part of the body often charged with norms and emotions related to sexgender and sexuality (Hunter et al., 2014). They also involve trying to exam the internal body through an orifice, and they both are often used in association with ultrasound technology to ‘see’ inside the body. The prostate exam is conducted by inserting a doctor’s finger into the patient’s anus and feeling the approximate size, shape and firmness/texture of the prostate, to see if it is hard or lumpy, possibly indicating cancer. This exam can be part of a process of diagnosing cancer, benign prostate hypoplasia or prostatitis. The bimanual pelvic exam is conducted by placing the one hand on the patient’s abdomen and inserting two fingers of the other hand into the vagina to feel the firmness of the cervix, the uterus’ shape, placement and texture, checking for cysts on it and the ovaries.

Both examinations are taught to all medical students and carried out at times by general practitioners, even though both are also standard exams for their respective specialties, urology and gynaecology. There are material-discursive differences, of course. For example, the prostate exam is often carried out in a regular examination room on a standard examination bed rather than in a specifically designed chair, which we will discuss later in this paper.

Our aim in studying these two exams together is to show that the pelvic and the prostate exam do much more than determine the health (or not) of an anatomical body part. They also combine with cultural understandings to produce and reproduce very different affective subjectivities for the doctors and the patients in practices of care.

At the particular medical education program in Sweden which we used as our case, students are taught the pelvic exam in learning sessions where they practice it on so called professional patients; volunteer cis females (someone who identifies as female and was assigned a female sex at birth) who use their own bodies to teach medical students doing the examination. This is offered at two occasions, during the second and fifth year. The first of these sessions focuses on teaching the exam step-by-step. Together, the teaching gynaecologist and the professional patients talked the students through the whole patient encounter, from calling in the patient from the waiting room, to talking with her, to how to position oneself as an examiner and how to move one’s hands, and even to when and where to look (both at the genitals, and when and how to make eye-contact with the patient). This is repeated during the final semester when the students again practice the exam on professional patients. At this second occasion, students also practice how to use a speculum, an instrument that opens the vaginal walls, used to facilitate many different procedures such as pap smear tests or insertion of an intrauterine device. This way of teaching the pelvic exam and to introduce it early in students’ education has been recognized as successful as it eases the students’ anxiety (Gleisner and Siwe, 2020; Siwe et al., 2007; Smith et al., 2015).

In contrast, at this medical education program, prostate exams are taught during clinical practice, especially during the fourth year when the students are placed in surgical clinics and wards. They may have observed and/or performed rectal examinations in other clinics, as well, since it is not only conducted for examining the prostate. But most rectal exams scheduled in the curriculum are done when practicing prostate exams. The students do the exam on actual patients at the clinic with meagre theoretical preparations. However, doctor-patient encounters at the urology clinic followed a well-established script: the patient initially being encouraged to narrate why he is there, followed by the doctor presenting a medical point of view, then conducting the medical examination or procedure, and ending with the doctor explaining possible findings and how to proceed. If a medical student is present, s/he will also examine the patient, contingent upon patient approval. Hence, these two intimate examinations are taught and conceptualized in different ways at this medical program.

Our primary material consists of interviews with medical students, observations in hospital settings focusing on prostate examinations, and observations of students practicing pelvic examinations with professional patients. Gleisner, who is trained in anthropology, spent three evenings at a gynaecological clinic when, all together, 16 students practiced pelvic examinations on professional patients. Gleisner followed a urologist for 2 days meeting prostate patients to get an insight of what a day at the urology clinic could look like and how a patient encounter could be carried out. During one of these days the urologist also supervised a medical student. Two urologists were interviewed about doing prostate examinations and supervising students, providing context and insight into their profession as well as teaching their profession to students. Gleisner also conducted in-depth, qualitative interviews with nine medical students, lasting approximately 1 hour each. The students studied their second, fourth or final year of medical school in Sweden (medical education in Sweden spans 5.5 years). Interview questions addressed learning to perform these two examinations, and in relation to that, how to approach patients, touching and examining patient bodies and the process of becoming a doctor. The limited empirical material is mitigated by the earlier research both authors have conducted on medical training, gynaecology and urology.

Johnson, a medical sociologist at a department of gender studies, has integrated this study into a wider, interdisciplinary programme of research on the prostate and has collaboratively shaped the direction of the analysis. The analysis is also contextualized against ethnographic work with midwifery training done for a previous study (Gleisner, 2013) and previous research involving observations of the gynaecological professional patient training compared with simulator training (Johnson, 2008; Dieckmann et al., 2019).

We conducted our analysis together, through close readings of the fieldnotes, attention to moments in the observations and interviews that indicated a disruption or affective response, and reflection on these notes against other studies of care and affective subjectivities. In our material, we consider feelings and emotions as findings (c.f. Roberts, 2015). We are writing about the feelings that doctors and patients express, and the feelings they are expected to have, drawing directly from ethnographic fieldwork (observations), of these exams in hospital settings and interviews with students and doctors. Bringing attention to feelings and emotions in students’ training elucidates how the prostate exam and the pelvic exam also become ‘gendered and gendering problems’ because, as we will show below, normative understandings about a patient and the body are entangled with how these exams are taught and carried out in medical care practice.

Thus, this study primarily relies on ethnographic observation and interviews with Swedish medical doctors and students, which limits the types of generalizations which can be drawn from a qualitative study of this sort, with what could be considered a small body of material. Additionally, our material is bounded by language (citations have been translated by the authors from Swedish) and must be contextualized as occurring in a tax-funded healthcare structure that provides universal healthcare for nominal cost at point-of-delivery. However, and in line with the ethnographic traditions of sociology, STS and anthropology which we work in, we feel the empirics presented here generate a space to reflect upon some situated, sexgendered aspects of how affective care is taught by and to health care professionals, how care for the patient’s understanding of what is normal is imagined, and how care for an individual patient’s integrity and modesty during the two intimate exams is discussed.

The study has followed the ethical guidelines of *the Swedish Research Council* for research with professionals about their profession or the learning of that profession. In line with the practices of our institution, ethics board approval was secured for the umbrella study within which this was placed.^4^ All participants granted informed consent, and written approval from the director of the medical program was procured. Even though the focus of this study is students’ learning and educational practices, both professional patients and clinical patients were present during observations. They were all informed about the study prior to observations and granted informed consent. No identifying information about patients was collected.

## Becoming doctors, becoming (dis)affected subjects

### The pelvic examination

The professional patient Linda is positioned in the gynaecological chair, partially reclined on her back and with her legs in stirrups. Fluorescent lamps light up the room and an additional lamp is directed towards her genitals. She is dressed in a hospital gown, blue with buttons down the front, and with long white socks to warm her. She has an extra pillow behind her head so that she can maintain eye contact with the students without straining her neck. The socks and the extra pillow are only used for these special occasions and not during regular patient examinations, the teaching gynaecologist explains. Two second-year students stand on either side of Linda and a third one is between her legs. The students seem nervous, eyes flickering, hands constantly moving as they have not yet learned how to position their bodies or where to look when meeting patients. In a firm voice, Linda tells the student in front of her to place his hand on her knee before adjusting the chair. ‘It makes me feel safe and not as if you are sending me through the ceiling’, she says. She tells him to raise the chair up a bit further and explains: ‘This is your working environment and you have to think about that. If you’re standing in a crooked position all day, it will cause pain in your back and shoulders’. She turns to the whole group when saying this and the students begin to laugh.

This scene is taken from observations of a training session where second-year students practiced doing the pelvic exam on professional patients. The medical doctor’s (and in particular, the medical students’) affective responses to the patient and the examination were fundamental to the development of professional patients in gynaecology examinations at the university hospital where our work was conducted (Siwe et al., 2007, 2013) as well as for gynaecology training programmes internationally (Underman, 2020). While mention may be made of how unpleasant it is for a female patient to be the body upon which a new student is learning the exam, literature about the use of professional patients also underlines that students probably think that their first pelvic exam is difficult, both physically and emotionally (Siwe et al., 2012; Underman, 2015; Wånggren et al., 2009). This is partly because the examination is largely based on the sense of touch, as one is examining interior parts of the body not visible without an ultrasound. It is hard to know if one is touching the right things and in the right way. But it is also imagined to be because one is examining the female genitals, and the medical student is positioned between the legs of a half-naked woman. This is presented as a normal cause of emotional stress. One does not normally find one’s self in that position, and that emotional stress is not only ‘normalized’ by the professional patient training, it is also recognized, legitimated and then the student is given tools by the professional patient and the instructor to deal with their emotions (c.f. Giuffre and Williams, 2000).

During observations of learning sessions with professional patients and in the interviews, students mentioned and expressed feelings of gratitude, of awe, fascination, a sense of pride and wonder at having done the exam ‘the first time’. ‘This is so cool!’, one of the second-year students said when watching the teaching gynaecologist point out the ovaries made visible through the ultrasound device. But she immediately corrected herself by saying to the students next to her ‘Right, don’t be so enthusiastic’. The students are taught to think of the female genitals as amazing, but also taught to not show this wonder, as doctors, during an examination; to consciously control their affective response as professionals. Yet, acknowledging the feelings evoked through the learning session was encouraged by the teaching gynaecologist. At the end of the learning session she asked how they (the students) felt. There was an unquestionably positive fascination of the female genitals and the conversation during the post-exam teaching session included remarks about everyone now wanting to become gynaecologists.

This intentional affective training produces positive feelings evoked through the training on the professional patients. Through the professional patient exams, the students are allowed to be ‘affective subjects’ and are taught how to professionalize that affect in a caring way which entangled respect, wonder and awe for the female genitals together with the stress and embarrassment of finding one’s self between the legs of a half-naked woman. Thus, through the learning sessions, there is both learning how to be affected as a professional subject and the expression of a collective responsibility for those being affected and cared for (c.f. Singleton, 2012).

### The prostate examination

The urologist Hanna, the fourth-year medical student David, and I (Gleisner) are sitting in Hanna’s office. There are a few minutes left before we need to go see the next patient. Hanna tells us about him: a man in his sixties experiencing urination problems. PSA-tests have shown increased levels lately. And his father had recently undergone surgery due to prostate cancer.

Just like with the previous patients, Hanna walks into the examination room first to ask the patient if David and I could participate. We are let in, just like with all patient meetings that day.

Hanna pulls out chairs for us and we sit down close to the patient. Hanna begins by asking him to narrate why he is there, at the urology clinic. He tells us about his worries, that he was afraid that he also has cancer, just like his father. Hanna listens to him, talks about his PSA-tests and then explains that she is going to examine the prostate, do an ultrasound and a biopsy. She instructs him how to position himself on the examination table.

While the patient pulls down his pants and underwear, and lays down on his side on the stretcher, with his back turned toward us, Hanna and David put on plastic aprons and gloves. Hanna sits down on a stool, puts lubricant onto her right index finger, rests her left hand on the patient’s hip and tells him that she is going to start examining. She gently inserts her finger into the patient’s rectum and then goes down on one knee trying to reach further in. Hanna later explains to us that she needs to do that sometimes because her fingers are so short. When David examines the patient after Hanna, she mumbles to him if he had felt ‘that’ and David hums in response. They change places again so that Hanna can do the ultrasound examination and the biopsy, assisted by a nurse who has entered the room.

Hanna slowly inserts the lubricated ultrasound probe. She points at the screen and explains to David and me the contours of the prostate visualized through the device. Without saying anything, she taps her finger at a dark spot that she apparently wants us to notice.

Throughout the patient encounters observed at the urology clinic, the urologist turned around while the patient undressed or dressed himself. This mirrors a strategy that was explicitly taught to the students when practicing pelvic exams on professional patients, the production of care for the patient’s personal integrity.

However, affect in the doctor and the patient encounter are taught and expected differently in the learning situations of these two exams. For example, rather than eliciting a ‘wow’ response in the medical students, like the female body did, examining the prostate rather provoked other kinds of feelings in describing the exam and the body. When asked during interviews if they remember doing the first pelvic exam, the students vividly described the shocking, fascinating or amazing experience it was, as described earlier. They could, in detail, recall the time and place for it as well as their own experiences, of course being reinforced by the specific learning sessions and that this was the first bodily exam they practice (except listening to hearts and lungs). When the fourth and final-year students were asked if they remembered their first prostate exams, their responses differed from the gynaecological exam on two points. Either that they did not recall it at all (‘It must have been during the placement in surgery/urology. . .’) or it was described in relation to teaching aspects, or rather the lack of teaching aspects, as in: ‘The supervisor just told me to insert my finger’ or ‘Now it’s your turn’ he said, ‘without any kind of instructions of how or what to search for’. One of the final-year students, however, described the experience of doing a prostate exam for the first time in relation to the patient’s body. She said,

I remember that I thought to myself ‘I have my finger in another person’s butt. This is so weird’. But it was over so quickly. And after you’ve done a couple of them, eventually it’s not a big thing.

When asked about describing the prostate examination compared to other examinations they learn, one of the fourth-year students said, ‘It’s not an exam that you choose to do’. When prompted to elaborate, he laughed and said, ‘Well, there are more enjoyable things’. He continued,

It’s a taboo thing. . . nothing you would do outside of the examination room. You do it in the role of being a doctor and then it’s okay, the normal boundaries disappear, and it feels relevant to do it. So yeah, it’s a special kind of examination but the more you do the less stigmatizing it gets. But now I also know what to look for when examining.

Another student also mentioned that there was a distinction in the frequency, or at least the perceived specialness of doing pelvic examinations compared to rectal examinations.

Pelvic exams are usually done at special clinics, while prostate exams are often done more ‘off the cuff’.’ *The student laughed a little uncomfortably*. That sounded horrible. Not ‘off the cuff’, really. . . but digital rectal exams are used for many different indications, and it isn’t just prostate palpations that can indicate them.

While the pelvic exam is almost exclusively discussed as a sensitive exam in literature (see Hilden et al., 2003; Siwe et al., 2013; Underman, 2011, 2020) as well as in the empirical material, the prostate exam is more ambiguous. It is given little attention in the students’ course literature, even though it is also described as ‘. . . one of the more intrusive examinations’ and that ‘men experience it as unpleasant’ (Damber and Peeker, 2012 [2006]). But the students are not advised during training on how to care for the patient’s experience as carefully as during the gynaecology courses.

Engaging with the concept of affective subjects employed in psychology, we can consider how during this moment of teaching a basic element of healthcare, the doctor and student were being affected by the patient – but not the patient-as-prostate, rather the patient as an entanglement of potential disease, age, fear, angst and stoic masculinity (Dakum et al., 2007; Patulny et al., 2017). The bodies they are examining are there because of a problem and the fear of disease, cancer and death is present in the expectations of those in the room, which, of course, makes them present and capable of producing affect.

Given that the patient is already at the doctor’s office because of a health concern and not generally for a standard check-up (rare in Sweden) or as a professional patient, the student may feel it is more imperative to maintain a disaffected demeanour in front of the patient than they would in a purely teaching situation. However, these sources of affect are not articulated during the teaching of the exam, they are merely ignored verbally and addressed through the choice of examination method and position. This non-recognition of the affect is (re)producing the stoic patient and the unaffected urologist – two subjectivities which have been reflected upon in sociological studies of prostate cancer practices (Dowsett, 2018; Perlman, 2018; Winterich et al., 2009). And apart from the affective state of the already worrying patient, the prostate exam, itself, does create affect, which needs care directed at it, in both the students and the imagined patients. As the example of what position a prostate patient is examined in, below, will show, there is a good deal of affective response to imagined sexual norms and taboos that are considered and cared for when dealing with prostate exams.

## Material aspects of the affective examination entanglements

Looking more closely at the material positionings of the patient and doctor bodies elucidates the emotional responses entangled with these exams. For example, material difference is found in the table/chair that the patient’s body is placed upon. Hereon we will explore the context-specific sites of care (Giraud and Hollin, 2016) and the material affordances of the patient and doctor bodies in the affective work of the care that is provided.

When entering an examination room at the urology clinic, one could not immediately see what kind of examinations were carried out in there, or even which clinic it was. In comparison, an examination room at the gynaecological clinic was unmistakably recognizable by the gynaecological chair dominating the room.

The gynaecological chair dictates the patient’s position and is designed for the gynaecologist’s needs. It provokes certain feelings attached to the patient’s exposed position (Börjesson et al., 2016; Edelstam et al., 2019). Some minor adjustments can be made, for example to the height of the stirrups. But it is primarily the patient who needs to adjust to the examination chair, rather than the other way around, an adjustment that the gynaecologist initiates by asking the patient to move further down into a position where the patient’s pelvis is tilted into a favourable position for the examination. Patients and doctors describe the position as if one is almost falling out of the chair (Ehrnberger et al., 2017). The height of the gynaecological chair could be altered depending on how tall the examiner is and whether they are standing or sitting on a stool.

Reflecting back over the scene from the pelvic exam, we would like to analyse something peculiar that brought the students to laughter during the training session. The professional patient who took on the instructor’s role not only taught and guided the students in doing the examination in a proper way but also cared for their experience of doing the examination as well as their role of becoming an examiner. The latter included very practical aspects of examining such as the working position. These inexperienced second-year medical students, who had so far only listened to hearts and lungs, saw and did their first bimanual pelvic examination and they had just been told how sensitive it is and the importance of caring for the patient’s feelings. Meanwhile, the woman in the gynaecological chair, half-naked and in a position that is often described as exposed and vulnerable, encouraged them to think about the ergonomics of their own working position. The irony of this redirection of care practices produced a discursive rupture and induced laughter.

In contrast to the gynaecological chair, the examination table/bed is not exclusive for urology, it is part of the standard equipment in many kinds of examination rooms. Nor is there one specific position for having one’s prostate examined; doctors may prefer different positioning of the patients. The patient could be in a forward-bending position, standing on the floor with hands on the examination table, or on hands and knees on the examination table, or, as depicted in the excerpt presented earlier, laying on his side. In all these different positions the examining doctor is positioned behind the patient, which inhibits eye contact. One of the urologists said,

You can’t see if the patients are in pain since they lay with their backs towards you. That makes this situation special. Otherwise you could just look up, but in this case, you have no idea what he is feeling.

Though this comment indicates that the urologist is aware of and trying to be responsive to what the patient is feeling, the constellation of material artefacts and bodies in the room prevents a visual indication of the patient’s experience. As the patient’s position is not predetermined by the examination table, and doctors may have different preferences for how to examine the patient, the doctors must explain to the patient how they should position themselves on the table each time the patient enters the examination room. A different urologist, who preferred the patients to be standing up and leaning over the table, explained,

I usually prefer them standing as it gives you a better notion of the prostate. But it is a bit tricky. It is a very. . . well, it is a difficult situation. That position is difficult for a man.

The position the doctor in this quote is referring to, when the patient has pulled down his trousers and bends over while the doctor is standing behind inserting a finger into his rectum, shows that it is not only what is examined that is sensitive but also how. This positioning of bodies brings with it associations of receiving anal penetration, which adds to the sensitivity of the situation. By being behind the patient, eye contact is prevented, or at least made difficult. This makes ‘easy’ a practice of avoiding eye contact, of looking away metaphorically and literally as the patient endures a procedure which is possibly both painful and uneasy, with sexual undercurrents that could be embarrassing (Winterich et al., 2009).

The students in the fourth and in the final year who had done the prostate examination also spoke of this difficult patient position and the benefits of having the patient laying on his side due to this. This was the position they had most often seen in practice. The most preferable position was thus negotiated in relation to the patients’ feelings of discomfort, rather than the doctor’s best option. And, as the scene of the urologist kneeling beside the examination table to get her finger further into the patient’s anus demonstrates, there is little concern for the ergonomically planned work environment in the urology clinic.

Hence, the urology exam positions the patient as the important factor in determining position – the urologist needs to contort their body into odd positions to feel the prostate when the man is laying on his side – while the gynaecological chair is used to position the patient into a way that allows for comfortable examination for the gynaecologist.

The material-discursive practices of the two exams do care through privacy or trust to varying degrees. They both employ understandings of discretion and the desire not to expose the patient, but also to different degrees. The gynaecological examination room has an additional lamp that could be adjusted as a spotlight directed at the patient’s genitals and a curtained-off changing room behind which the patient can undress, creating privacy and also articulating when and where the patient is observed by the examining doctor. The students were told not to look at the patient undressing or when climbing into the chair, but to wait for the patient to be in the chair; that is when they should look. When the teaching gynaecologist and the professional patient initially showed how to perform the exam, they both encouraged the students to look. ‘Come closer so you can see’, the gynaecologist said. The students slowly approached, but their unease seemed to switch into amazement when the gynaecologist used the speculums to open the vaginal walls so that the students could see the cervix.

In contrast, during the prostate exam, the lights were dimmed, as it helps to see the ultrasound image more clearly. When asked about this, the urologist said of the body on the examination table, ‘there is nothing to see’. When doing a prostate examination, there is nothing to observe on the outside of the body. What is observed is first felt by the inserted fingers and then digitally visualized through the ultrasound device that shows the contours, size and shape of the prostate and possible abnormal findings.

There is a materialized irony of this in the examinations, given that the gynaecological chair produces an exposed and vulnerable body even as that body is protected in the actual care practices of the pelvic exam. Quite the opposite occurs in the prostate examinations, where the darkened room and choice of examination position reduces the patient’s exposure, even as the practices and people examining him do not discursively label this as ‘care’ for his feeling of modesty and integrity. That ‘there is nothing to see’ when doing a prostate exam does not mean that there is nothing to be exposed. We suggest that dimming the lights can be read as a way of caring about the patient and his exposed and vulnerable position, in a way that is not considered (necessary) for the gynaecology patients because care for their integrity is done in other practices.

The material aspects in the context-specific sites of urology and gynaecology contribute to discussions on different affective subjects produced in medical care practice, what is cared for and what is dismissed. As Giraud and Hollin (2016) show, care in practice is continuously done between practitioners, the ones being cared for, the material, but also its history and context. The material world of gynaecology creates and exposes a tenuous subject position for the female body. The material world of urology allows for a protected and respected male body to be examined. We will discuss historical trajectories of these two fields and their impact on affective subjectivities, below.

## Discussion

We began this article by introducing aspects of care that appear to differing degrees around intimate exams and which we want to continue discussing, with a concern for care as a medical practice; that care is much more complex than the mere practice of providing an exam in the name of health care; care allows us to ask questions about subject positions; care is entangled with assumptions about the patient experiences and influenced by values attributed to sexgender, sexuality, age and health.

Through a perspective on care with a concern for the material-discursive production of intimate exams, the article has detailed medical care practices that create specific patient and doctor affective subjectivities. It has done so by looking into how medical students are taught and reflect upon such an intimate examination as the digital rectal examination of the prostate and comparing and contrasting this with another intimate examination, the bimanual pelvic examination, asking who is being cared for (Singleton, 2012) and reading bodies as material-semiotic actors (Haraway, 1991) with attention to affect and the subject (Roberts, 2015).

The students were aware that both of the exams were producing emotional responses (affective subjectivities) in themselves as care givers and potentially in the patients. Yet, as we have shown, there are differences in how the meeting between the prostate exam patient and doctor and how the pelvic exam patient and doctor is taught to students. We have been struck by the production of caring and respectful gynaecologists who speak to the patient, look her in the eye, prepare her for the exam, explain what will happen and what the doctor is about to do, all the time working with an understanding of the patient as a sensitive subject who may be experiencing this as an unpleasant exam and trying to make it slightly less unpleasant. This became even more striking when we saw how it contrasts with the scene in the prostate exam, and the production of a less affective subject, one which is less concerned with emotions, or with the affect of the patient.

Hence, the evening courses for the students with professional patients were meant to provide insight into the patient’s experience. The way the pelvic exam is embedded in clinical work practices engages discretion to make the patient feel comfortable and secure. In contrast, even though the urologist would turn her back to let the prostate patient undress, and the students reflected on the affective nature of the prostate exam to some degree, the structural elements that produced the affective gynaecology patient were missing in the prostate exam.

We suggest that there are historical and structural explanations for this. The gynaecology teaching programme we observed was initiated and run by an older, well-established gynaecologist at the university hospital who had, as a young medical student, been shocked by the use of unknowing, anaesthetized female patients as bodies to train the gynaecological exam upon.^5^ The professional patient programme was developed as a way of improving the care women can receive. This resonates with the reasons for using professional gynaecology patients in many other teaching hospitals and medical schools (Underman, 2011, 2015), and reflects a concern for the patient experience that may be traced to the impact the women’s health movement has had on medical training and practice (Murphy, 2012; Tuana, 2006).

The teaching and practicing of the urology exam does not seem to address the potential for embarrassment or discomfort with the same articulated practices or structural/clinical routines, even though established urologists interviewed for a different part of this study indicated that they have, in the course of their career, adapted their patient-doctor demeaner to address patient discomfort and embarrassment (Johnson, 2021). Men’s health care has not experienced the same political pressure to adapt to patient demands for changed care practices or epistemological critique, which, as Epstein notes, may reflect the political untenability of men to organize around their identity as, specifically, men (Epstein, 2007). Though, we realize that this has not stopped some men from trying, and that patient/disease-specific activism would appear to be coalescing around particular diseases associated with male bodies, like prostate cancer.

The different historical developments of the fields or urology and gynaecology, the various political pressures they have felt from activists inside and outside the medical establishment, and the way the patients they treat are imagined, produce different affective encounters.

We also note that we see that the structures around teaching and providing care are reproducing gendered understandings of the patient. We have observed variations of what, in their extreme, is a sensitive, sexually vulnerable woman easily embarrassed or triggered by an exam of her reproductive tract (c.f. Murphy, 2012) and a strong, insensitive man who just gets the job done (c.f. Dowsett, 2018). These are also reproducing gendered understandings of the professional performing the care provision. Gynaecology is still, in Sweden, a very female dominated field (67%) and urology is male (only 16% women) (The Swedish Medical Association, 2013). While the students we followed were a mix of male and female, the exams they were learning were connected to single sex dominated fields.

Given the observations about the female dominance of care and emotional work, especially in health care (Duffy, 2011; Meyer, 2000), it is perhaps no surprise that the field that is populated by female bodies (gynaecology) is the one that discursively embedded the exam in articulated work to produce sensitive, caring practice. This reproduces a sexgendered understanding of the future gynaecologist. Likewise, even though the doing of care and discretion were observed in the urology exam (as mentioned above) this field is not reproducing female sexgendered practitioners who are expected to be ‘caring’ in the same way, and is, rather reproducing a male urologist ideal (even if the body actually doing the care happens to be female), one which the literature on care would suggest may be less interested in vocalizing the emotional work of care. And we emphasize: this is not to say that urologists do not care nor do emotional work; they do. However, articulating this aspect of their professional identity by talking about it and discursively reinforcing it in the teaching was not something we observed to the same extent.

The teaching of these two examinations in the medical education of doctors contributes to these different subject positions for the patients and doctors. Gynaecology students are taught by professional patients and are actively encouraged to think about their own and the patient’s emotions, background and past experiences, and also how to express themselves during the patient encounter. The urology students are taught by practicing urologists during actual examinations.

Looking into teaching situations also draws out the collective aspects of medical students learning a profession (Gleisner, 2013) and learning the ‘proper’ emotional response (Fineman, 2005; Hochschild, 1979) in the examining situations. As we watched students learning the prostate and pelvic exams, we heard them expressing affect in very different ways and observed very specific types of subjects being performed. The prostate exams produced affected medical professionals who were displaying affective neutrality. In the gynaecological exams, we saw the production of affected subjects who expressed awe and respect for the body they were confronted with. The bodies being examined were expected to perform the patient in normative ways which reproduced their sexgendered status and norms and values related to that (often narrowly and heteronormatively) sexgendered subject.

Using a professional patient will produce a very different environment for teaching and learning than practicing an intimate exam on a patient who is there because of concerns and worry about their health. And we wonder whether the overwhelming presence of cancer fear is relevant to consider in the urology exam, but also if the average age and status of a urology patient are useful in explaining these differences. This is something which Gleisner has analysed elsewhere with her interlocuters in the field (Gleisner and Siwe, 2020). While it is not the focus of this article, we suggest that these different circumstances are also related to the different imaginaries of the patient’s needs and expectations, and are impacted by the historical developments of the fields and the sexgendered subjectivities imagined by the doctors about the patients (c.f. Bajeux, 2020; Björkman and Persson, 2021). And, as Gleisner notes, it may be beneficial to think about how the use of professional patients for prostate exams – something which does occur at some urology departments in the USA – could be engaged more (Gleisner and Siwe, 2020).

To conclude, we want to return to the knots of material-discursive entanglements in practices of care, that we, inspired by Haraway (1997) and Roberts (2015), have tried to loosen and discuss. The affective patient and doctors are produced in material-discursive constellations of human and non-human actors, of tables and tools as well as naked bodies and those wearing hospital scrubs. The body of a subject becomes particularly relevant in medical examinations, as its entanglements with social characteristics, affective responses and relational intra-actions with other people and things in an examination room produce the object of examination, the particular anatomical body which the subject becomes during the exam. But, as our examples show, that anatomical body is much less flesh and blood and much more affective responses and relational intra-actions than a medical textbook would suggest. And the ways medical students are taught to approach the patient’s body in particular exams are an important part of producing affective subjects.

Finally, the ‘affective subjects’ that are being cared for demonstrate the entangled knots of affect, sexuality, masculinity, femininity, patienthood and care in the practices of pelvic and prostate exams. Affective subjects are done in relation to each other throughout the care elements of the exam – producing authority for the doctor by a neutral, emotionally unaffected authority in one case and a sensitive and respectful authority in the other.

## Notes

1. PSA, short for Prostate-specific antigen, is a substance produced by the prostate and is measured through a blood test. A high level may indicate cancer, prostatitis or enlarged prostate.

2. We use sexgender to indicate that we understand the concept as a categorizing tool that references and actions material-discursive, intra-acting elements of the biological body and the social structures, norms and values. This term recognizes that what is understood as a boundary between the biological and the social is fluid and often arbitrary.

3. In both cases, the instructor was a cis woman, the observer (Gleisner) was a cis woman, and the students were cis men and cis women. The patients were cis men in the urology exams and cis women in the gynaecological course.

4. The decision of the Regionala etikprövningsnämnden I Linköping, avdelning för prövning av övrig forskning, dnr 2016/167-31.

5. Personal communication. See also Shrestha et al. (2010) and Wijma et al. (1998).
